# Low creatinine levels in diabetes mellitus among older individuals: the Yuport Medical Checkup Center Study

**DOI:** 10.1038/s41598-021-94441-9

**Published:** 2021-07-26

**Authors:** Saori Kashima, Kazuo Inoue, Masatoshi Matsumoto

**Affiliations:** 1 Environmental Health Sciences Laboratory, Graduate School of Advanced Science and Engineering, Hiroshima University, 1-5-1 Kagamiyama, Higashi-Hiroshima, Hiroshima 739-8529 Japan; 2 Department of Community Medicine, Chiba Medical Center, Teikyo University School of Medicine, 3426-3 Anesaki, Ichihara, Chiba 299-0111 Japan; 3 Department of Community-Based Medical System, Graduate School of Biomedical and Health Sciences, Hiroshima University, 1-2-3 Kasumi, Minami-ku, Hiroshima, 734-8551 Japan

**Keywords:** Endocrine system and metabolic diseases, Epidemiology, Diseases, Medical research, Geriatrics

## Abstract

ORIC ID: 0000-0002-3401-8191. It is unknown whether the interrelationship between diabetes and muscle loss is affected by ageing. Therefore, the serum creatinine levels, an indicator of muscle mass, were compared between older people with diabetes and those without diabetes, using a cross-sectional dataset from the Yuport Medical Checkup Center Study. We classified 6133 participants without kidney dysfunction into three age-groups: early-elderly (65–69 years), middle-elderly (70–74 years), and late-elderly (≥ 75 years). The association between diabetes and the lowest creatinine level, defined as less than or equal to the 25 percentile of serum creatinine, was evaluated in each age group, by calculating the odds ratio (OR) using logistic regression. Serum creatinine levels increased with ageing in the participants, and these trends were markedly observed in the non-diabetic group. Late-elderly people with diabetes were significantly more likely to have low creatinine levels than those without diabetes, with adjusted ORs 2.50 (95% CI 1.99–4.50) in men and 2.88 (95% CI 1.47–5.64) in women. Ageing modified the effect of their diabetes status towards a lower creatinine level (p for interactions between the diabetic status and age-groups were 0.01 in men and 0.05 in women, respectively). Ageing may thus accelerate the loss of muscle mass in people with diabetes.

## Introduction

The increasing number of older people have prospected globally, and the proportion of people aged 65 years or older is estimated to reach nearly twice as those from 2015 (elderly rate 12%) to 2050 (22%)^1^. Ageing is accompanied by major changes in body composition, including a progressive decrease in muscle mass, muscle strength, and its quality^2^; a decrease in glucose tolerance^3^; and an increase in the risks of chronic disease^4^. With an increasing number of older people, the prevalence of diabetes is expected to increase globally. For example, the prevalence of diabetes among people aged 75–79 years will increase from 1.4% in 2019 to 20.5% in 2045^5^.

In these circumstances, the World Health Organization launched a global strategy for healthy ageing in 2016^6^. Physical activity and maintenance or increase in muscle mass were addressed as significant determinants of “active ageing”^7^ and had a major role in reducing morbidity later in life^8,9^. Previous studies reported that low muscle mass is associated with an increased risk of type 2 diabetes and other cardiometabolic diseases^10–12^. Other studies have reported associations, in which people with diabetes lose muscle mass and strength more than those without diabetes^13,14^. Because of the lack of available data, the association between the diabetes status and the level of skeletal muscle mass in the late-elderly population i.e. in people aged 75 years or older is still unclear. Moreover, it is unknown whether the interrelationship between diabetes and muscle loss is affected by ageing. Although the management of diabetes for older adults should be tailored to the individual’s unique physical and social situations^15^, reliable information associating diabetes and ageing is scarce.

Although measuring the amount of skeletal muscle mass in a large population is difficult, serum creatinine levels indirectly reflect the amount of muscle in people whose kidneys function “normally”^16^. Japan has been at the forefront of a super-ageing society^17^, with a sufficient amount of data gathered on diabetes among older people through health check-up programmes. Therefore, we compared the serum creatinine levels between older people with diabetes and those without diabetes by using a large cross-sectional dataset from the Yuport Medical Checkup Center Study and evaluated the role of ageing in the interrelation between diabetes and serum creatinine levels.

## Results

Among the 6133 study participants, the mean age of men was 69.8 years (standard deviation, SD: 4.3) and of women was 69.4 years (SD: 3.8). Table 1 shows the characteristics of the study participants according to the three age-groups. A total of 438 men (15.0%) and 315 women (9.8%) were diagnosed as diabetes. The prevalence of diabetes in men were similar across age-groups; however, those in women were significantly different across age-groups (8.6%, 9.7%, and 17.2%, respectively). The mean fasting plasma glucose (FPG) levels did not change across age-groups in men, but these levels increased in women with ageing. The estimated glomerular filtration rates decreased with ageing, and the creatinine levels increased in the late-elderly groups in both men and women.

**Table 1:** Basic characteristics of the 6133 subjects according to the three age-groups with a normal creatinine level (< 115 μmol/l for men and < 106 μmol/l for women).

	Total (n = 6133)	Age group			
		65–69 years (n = 3522)	70–74 years (n = 1853)	≥ 75 years (n = 758)	p-value^‡^
**Men**					
N, persons	2923	1623	868	432	
Diagnosed as diabetes, n person*	438 (15.0)	243 (15.0)	127 (14.6)	68 (15.7)	0.871^§^
Body mass index, kg/m^2^	23.2 (2.8)	23.5 (2.7)	23.1 (2.8)	22.6 (2.8)	< 0.001
Fasting plasma glucose, mmol/l	5.8 (1.2)	5.8 (1.3)	5.8 (1.1)	5.9 (1.2)	0.353
Hemoglobin A1c, %	5.7 (0.9)	5.7 (0.9)	5.7 (0.8)	5.7 (0.9)	0.348
[Hemoglobin A1c, mmol/mol]	[38.8 (9.3)]	[38.7 (9.6)]	[38.7 (8.7)]	[39.4 (9.6)]	0.372
Creatinine, μmol/l	74.4 (12.0)	73.7 (11.7)	74.6 (12.0)	76.8 (12.9)	< 0.001
eGFR^#^, ml/min/1.73 m^2^	68.9 (12.9)	71.2 (12.9)	67.6 (12.2)	62.9 (12.4)	< 0.001
Cardiometabolic diseases, n person*^,†^	245 (8.4)	133 (8.2)	79 (9.1)	33 (7.6)	0.616^§^
**Women**					
N, persons	3210	1899	985	326	
Diagnosed as diabetes, n person*	315 (9.8)	163 (8.6)	96 (9.7)	56 (17.2)	< 0.001^§^
Body mass index, kg/m^2^	22.9 (3.1)	22.9 (3.1)	22.9 (3.0)	22.9 (3.3)	0.95
Fasting plasma glucose, mmol/l	5.6 (1.2)	5.5 (1.0)	5.6 (1.2)	6.0 (1.7)	< 0.001
Hemoglobin A1c, %	5.7 (0.8)	5.6 (0.8)	5.7 (0.8)	5.9 (1.0)	< 0.001
[Hemoglobin A1c, mmol/mol]	[38.8 (8.7)]	[38.4 (8.2)]	[38.9 (8.7)]	[40.8 (11.1)]	< 0.001
Creatinine, μmol/l	55.3 (9.6)	54.6 (9.2)	56.0 (10.0)	57.3 (10.3)	< 0.001
eGFR^#^, ml/min/1.73 m^2^	62.4 (12.7)	64.3 (12.5)	60.7 (12.5)	56.6 (11.8)	< 0.001
Cardiometabolic diseases, n person *^,†^	221 (6.9)	113 (6)	74 (7.5)	34 (10.4)	0.008^§^

Data are expressed as mean (standard deviation) or * number (%).

*eGFR* estimated glomerular filtration rate.

^†^ Number of persons who have a history of at least one chronic disease, including hypertension, hyperlipidaemia, cerebral stroke, myocardial infarction, and angina pectoris based on self-reporting.

^‡^ p-value was calculated using one-way analysis of variance.

^§^ p-value was calculated using the χ^2^ test.

^#^ eGFR was calculated by following equations: 194 × serum creatinine^−1.094^ × age^−0.287^ for men and 194 × serum creatinine^−1.094^ × age^−^^0.287^ × 0.739 for women, and adjusted by body-surface area.

**Figure 1:**
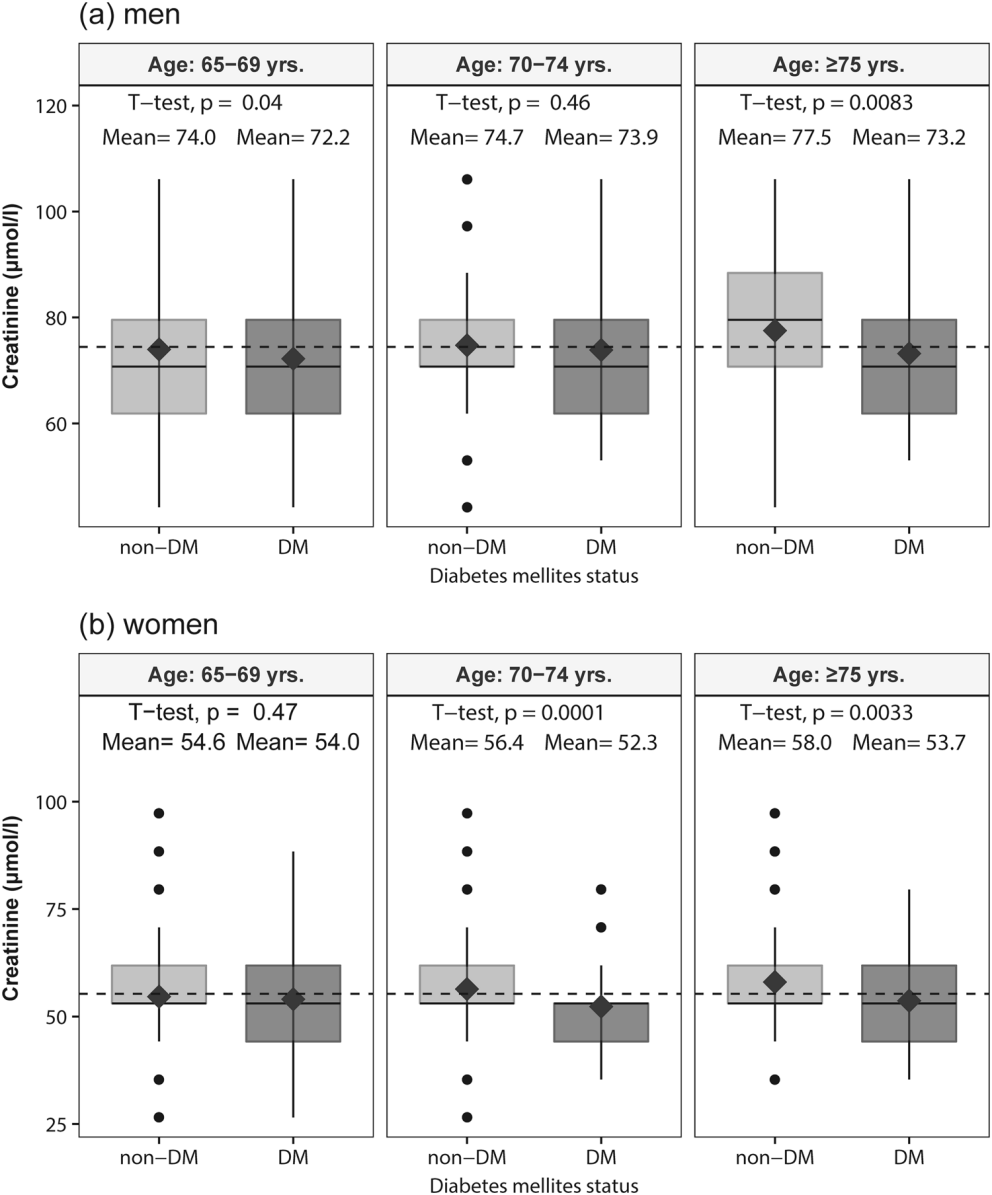
Boxplot of the creatinine levels across age-groups without diabetes (n = 5380) and those with diabetes (n = 753). The dark grey diamonds represent the mean serum creatinine levels. The bottom and top of the box represent the 25th and 75th percentiles, respectively, and the band near the centre of the box is the 50th percentile (median). ‘Whiskers’ represent the maximum and minimum that extend 1.5 times the inter-quartile ranges from the box edges. The vertical dashed line represents the mean creatinine across all participants by sex. *DM* diabetes mellitus.

Figure 1 shows the mean creatinine levels across the three age-groups of those without diabetes and those with diabetes. In men and women of all age-groups, the mean creatinine levels tended to be lower in participants with diabetes than in those without diabetes, and significant differences were observed in men belonging to the early- and late-elderly groups, and in women belonging to the middle- and late-elderly groups. Compared with those in the early- and middle-elderly groups, the differences in the mean creatinine between people without diabetes and those with diabetes were higher in the late-elderly group (4.3 μmol/l in both men and women).

**Figure 2:**
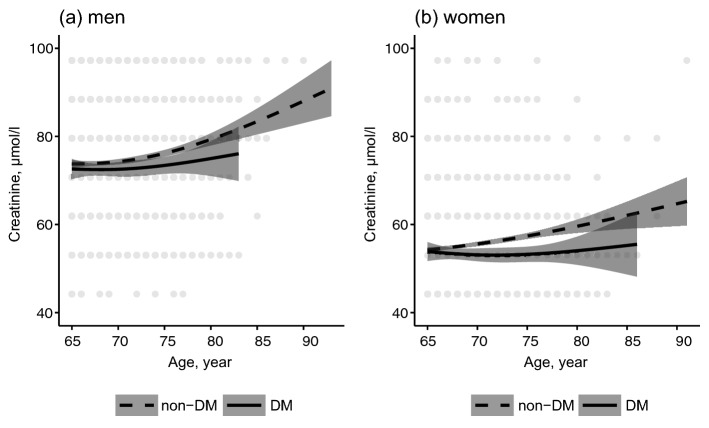
Linear regression line for creatinine levels against age stratified by diabetes status in (**a**) men and (**b**) women. The linear regression line with the natural spline function with 2 degrees of freedom was estimated according to the diabetes status, and the shaded areas indicate a 95% confidence interval. The oldest age was 83 years in men with DM, 86 years in women with DM, 93 years in men without DM, and 91 years in women without DM. *DM* diabetes mellitus.

Figure 2 shows the linear regression line for creatinine levels against age stratified by diabetes status. The creatinine levels were elevated with an increase in the age, and these trends were markedly observed in the non-diabetic group. Compared with people without diabetes, the increased levels of creatinine in people with diabetes were low in men and women. The oldest age was lesser in people with diabetes (men: 83 years and women: 86) than in people without diabetes (men: 93 years and women: 91).

**Figure 3:**
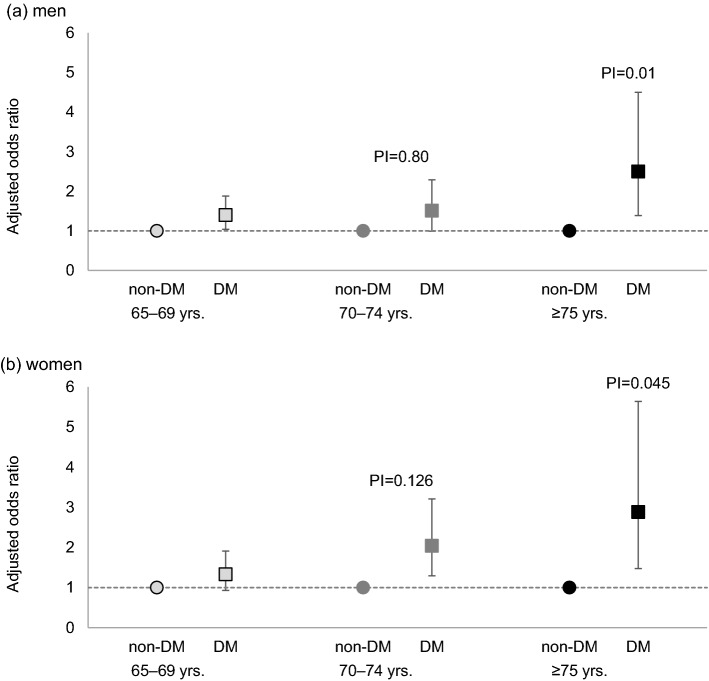
Adjusted odds ratios for low creatinine levels compared between participants without diabetes (n = 5380) and those with diabetes (n = 753) in men and women. The model was adjusted for age and body mass index. The low creatinine levels were defined as those which were less than the 25 percentile (25 percentiles were 61.9 μmol/l in men and 53.0 μmol/l in women, respectively). *DM* diabetes mellitus, *PI* p-value for the interaction of the diabetes status and age-groups.

Figure 3 shows the adjusted odds ratios (ORs) with 95% confidence intervals (CIs) for the lowest quartile of creatinine levels compared between participants without diabetes and those with diabetes by age group. In men and women, the highest ORs were observed in the late-elderly group, and age- and body mass index (BMI)-adjusted ORs were 2.50 (95% CI 1.99–4.50) in men and 2.88 (95% CI 1.47–5.64) in women. Statistically significant interaction terms between the diabetes status and age were observed in the late-elderly group. This means that the effect of diabetes status on the creatinine levels was different across age-groups, which is more prominent in older age.

## Discussion

This study used a large cross-sectional dataset comprising people 65 years and older and showed that those with diabetes had a higher prevalence of lower creatinine levels, which indirectly represented the amount of their muscle mass, compared to those persons without diabetes. Further, this association was markedly seen in the late-elderly group (75 years or older) than in the less aged groups. Thus, ageing may accelerate the cyclic interaction between diabetes and muscle loss.

**Figure 4:**
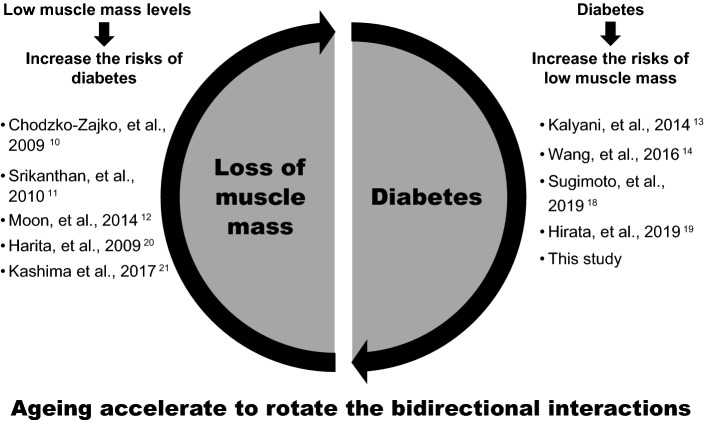
The bidirectional interactions between diabetes and loss of muscle mass with ageing effects.

Based on the findings of this study and previous literature referenced, we suggest a vicious cycle between diabetes and muscle loss: from diabetes to the loss of muscle mass and vice versa (Fig. 4). In these bidirectional interactions, the right pathway is that diabetes potentially promotes the loss of muscle mass and strength^13,14^. The findings of this study support those of the previous studies. Other studies in Japan reported that hyperglycaemia in non-obese patients with type 2 diabetes is the cause of low muscle mass levels^18^. Furthermore, the pathway in which hyperglycaemia induces skeletal muscle atrophy via the proteins WWP1/KLF15 were reported in an animal-based study^19^. In addition to these previous studies, this study showed that the association was prominent in the late-elderly group (age ≥ 75 years) in both men and women.

The left pathway in the bidirectional interactions is that low muscle mass levels increase the risk of diabetes^10–12^ (Fig. 4). Previous Japanese studies also reported that low creatinine levels were associated with an increased risk of diabetes in Japanese men^20^ and women^21^. In this study, we showed that the interactive associations between diabetes and loss of muscle mass were different across the three age-groups, and the association was markedly observed in the late-elderly group.

Ageing brings about changes in body composition, such as a decrease in glucose tolerance^3^, and an increased risk of diabetes^4^. Additionally, ageing decreases physical activity and functionally reduces muscle mass, muscle strength, and quality^2^. The mean daily step count of the middle-elderly people decreased by 18% from those of the young people (20–64 years), and this decreased by 41% in the late-elderly people in Japan^22^. Although the functional role of ageing in the association between diabetes and the loss of skeletal muscle is still to be explored, ageing may be a driving force for accelerating the proposed vicious cycle (Fig. 4). The growing need for tailored diabetes self-management is required for elderly people, and ageing, which increases the impact of diabetes on the loss of muscle mass, should be considered.

Because of the cross-sectional nature of this study, we were not able to assess the effect of diabetes control on serum creatinine levels. Good control of plasma glucose levels protects against the progression of both diabetic nephropathy and muscle loss. Protection of the kidneys decreases the creatinine levels while that of muscle mass increases it. The overall effect of diabetes control on creatinine levels is unclear and should be confirmed in future longitudinal studies. Furthermore, the characteristics of the baseline participants differed between men and women. For example, FPG and hemoglobin A1c (HbA1c) levels increased with age in women, but this trend was not observed in men. In addition, the age distribution was different between men and women. The proportion of women aged ≥ 75 years was 10.2%, but in men it was 14.8%. Thus, attention should be paid when generalising our results to other populations with different age and gender compositions.

This study has several limitations. Firstly, the study participants voluntarily participated in the health screening programme. Thus, they may represent healthier people than the general older population. In addition, there may be a survival effect, in that more people with diabetes with renal failure may have died at an earlier age. This phenomenon potentially could have reduced the proportion of very old people with diabetes with higher creatinine levels, while increasing the proportion of those with lower creatinine levels. Secondly, creatinine levels indirectly reflect the amount of muscle in the body when the kidneys are functioning “normally”, along with normal protein intake^16^. In this study, we attempted to resolve this issue by excluding subjects with a high creatinine level. Thirdly, glomerular hyperfiltration, which might have contributed to lower serum creatinine levels due to pre-diabetes or diabetes, might have affected the results of this study. Hyperfiltration has been reported to be related to a physiological decline in renal function during the process of ageing in diabetes^23^. However, another study reported a decreasing prevalence of hyperfiltration with increasing age in people with type 2 diabetes^24^. Thus, the effects of this bias might be limited in our findings of age-related differences in creatinine levels between people with and without diabetes. Fourthly, due to the cross-sectional nature of this study, we could not discuss the causality and the pathway between diabetes and the loss of muscle mass. Further studies should evaluate the role of ageing in the bidirectional interactions between diabetes and loss of muscle mass. Fifthly, we could not obtain data on confounders, other than sex, BMI and history of cardiometabolic disease, so residual confounders such as comorbidity, including frailty and cognitive dysfunction, lifestyle, including physical exercise and smoking status, and concomitant medications should be considered in further studies. Finally, we used creatinine level as a surrogate marker of body muscle mass. Creatinine levels have been used for this purpose in various studies^25–27^, but they do not directly show the amount of muscle mass. To improve the precision of our results, future studies utilising a direct measure of muscle volume should be conducted.

Since life expectancy is increasing globally, the population of older people with diabetes will increase. Ageing may accelerate the loss of muscle mass among people with diabetes, which was supported in this study. Along with this, decreasing muscle mass increases the risk of frailty or sarcopenia^13^. To achieve successful ageing, diabetes control, and increasing/maintaining skeletal muscle mass are essential.

## Methods

### Study subjects

This was a cross-sectional study, and the dataset of this study was derived from the health screening programme implemented by the Yuport Medical Checkup Centre in Tokyo^28^. A total of 34,303 persons voluntarily underwent a health check-up during the study period between April 1998 and March 2006. For the study, we selected 6215 participants, who were aged 65 years or older and had no missing values for creatinine, FPG, and HbA1c. We used the data for each participant during their first visit for their health check-up. To eliminate those with kidney dysfunction, 82 subjects whose creatinine levels were not within the normal reference range (≥ 115 μmol/l for men and ≥ 106 μmol/l for women) or whose estimated glomerular filtration rate (eGFR) was less than 15 indicating end-stage kidney disease were excluded. Finally, 6133 persons (2923 men and 3210 women) aged between 65 and 93 years were enrolled. All the check-up procedures were performed in the same manner at the Centre following facility guidelines.

### Diagnosis of type 2 diabetes

Type 2 diabetes was defined either as known diabetes, FPG ≥ 7.0 mmol/l, and/or HbA1c level ≥ 6.5% (47.5 mmol/mol) according to the current criteria for diabetes by the American Diabetes Association (ADA)^29^ and the Japan Diabetes Society (JDS)^30^. Known diabetes was identified when the presence of diabetes was reported by the participants at a medical interview irrespective of their FPG or HbA1c levels. FPG and HbA1c in a blood sample were obtained at the Centre’s laboratory after a participant fasted overnight, and these were measured using a Toshiba TBA-40FR Autoanalyser (Toshiba Medical Systems, Tokyo, Japan). The JDS value of HbA1c was converted into the National Glycohemoglobin Standardization Program (NGSP) units by using the following equation “in the range of JDS values ≤ 4.9%: NGSP (%) = JDS (%) + 0.3%, in the range of JDS values 5.0%–9.9%: NGSP (%) = JDS (%) + 0.4%, and in the range of JDS values ≥ 10%, NGSP (%) = JDS (%) + 0.5%”^31^.

### Creatinine

Serum creatinine levels were measured using enzymatic methods (reagents supplied by Mitsubishi Kagaku Iatron Inc., Tokyo, Japan).

### Statistical analysis

Since creatinine levels differ between sexes, all analyses were conducted separately in men and women. First, we classified the study participants into three age-groups: 65–69 years as early-elderly, 70–74 years as middle-elderly, and ≥ 75 years as late elderly. Analysis of covariance (ANCOVA) tests were used to compare the mean of each value across the three age-groups, and χ^2^ tests were used for analysis of the count data. Second, we described the characteristics of the study participants according to these three age-groups. We then compared the creatinine levels between people without diabetes and those with diabetes according to each age group by the *t* test. Third, we classified the participants according to their diabetes status and evaluated the trend of creatinine levels based on age. In this analysis, age was treated as a continuous variable and was entered into the linear regression model with a natural spline function with 2 degrees of freedom. Finally, to evaluate the association of the diabetic status with the occurrence of the lowest creatinine levels in each age group, we calculated the OR and 95% CI of the lowest creatinine level in people with diabetes compared to those without diabetes, using logistic regression. The lowest creatinine level was defined as that when a person’s creatinine level was less than or equal to the 25 percentile. The inter-quartile range of creatinine levels was 61.9–79.6 μmol/l in men and 53.0–61.8 μmol/l in women, respectively. In the model, we entered the age and BMI, which was calculated as weight (kg) divided by height square (m^2^) as potential confounders. We also evaluated the statistical interaction between the diabetic status and age-groups at a significance level of 0.10, by including an interaction term in the model^32^. R version 4.0.2 software (R Foundation for Statistical Computing, Vienna, Austria) and SPSS software (version 26.0J, IBM Corp., Armonk, NY, USA) were used for the analyses. A p-value of less than 0.05 was considered statistically significant.

### Ethics declarations

This study was performed in line with the principles of the Declaration of Helsinki. This study was approved by the institutional review board of the Teikyo University School of Medicine (No. 15-205) and Hiroshima University (No. E-1999).

### Consent to participate

For anonymous participation in epidemiological research, informed consent was obtained at every checkup.

## Data Availability

The data that support the findings of this study are available from the corresponding author and authors upon reasonable request.

## References

[1] United Nations, Department of Economic and Social Affairs, Population Division. *World Population Prospects 2019 Highlights *(*ST/ESA/SER.A/423*) (The United Nations, 2019).

[2] Cruz-Jentoft AJ, Sayer AA (2019). Sarcopenia. Lancet.

[3] Basu R (2003). Mechanisms of the age-associated deterioration in glucose tolerance: Contribution of alterations in insulin secretion, action, and clearance. Diabetes.

[4] Sakurai T (2010). Age-associated increase in abdominal obesity and insulin resistance, and usefulness of AHA/NHLBI definition of metabolic syndrome for predicting cardiovascular disease in Japanese elderly with type 2 diabetes mellitus. Gerontology.

[5] International Diabetes Federation (2019). IDF Diabetes Atlas.

[6] World Health Organization (2017). Global Strategy and Action Plan on Ageing and Health.

[7] Bauman A, Merom D, Bull FC, Buchner DM, Fiatarone Singh MA (2016). Updating the evidence for physical activity: Summative reviews of the epidemiological evidence, prevalence, and interventions to promote "active aging". Gerontologist.

[8] Crimmins EM (2015). Lifespan and Healthspan: Past, present, and promise. Gerontologist.

[9] Kalache A, Aboderin I, Hoskins I (2002). Compression of morbidity and active ageing: Key priorities for public health policy in the 21st century. Bull. World Health Organ..

[10] Chodzko-Zajko WJ (2009). American College of Sports Medicine position stand. Exercise and physical activity for older adults. Med. Sci. Sports Exerc..

[11] Srikanthan P, Hevener AL, Karlamangla AS (2010). Sarcopenia exacerbates obesity-associated insulin resistance and dysglycemia: Findings from the National Health and Nutrition Examination Survey III. PLoS One.

[12] Moon SS (2014). Low skeletal muscle mass is associated with insulin resistance, diabetes, and metabolic syndrome in the Korean population: The Korea National Health and Nutrition Examination Survey (KNHANES) 2009–2010. Endocr. J..

[13] Kalyani RR, Corriere M, Ferrucci L (2014). Age-related and disease-related muscle loss: The effect of diabetes, obesity, and other diseases. Lancet Diabetes Endocrinol..

[14] Wang T (2016). Type 2 diabetes mellitus is associated with increased risks of sarcopenia and pre-sarcopenia in Chinese elderly. Sci. Rep..

[15] Kirkman MS (2012). Diabetes in older adults. Diabetes Care.

[16] Schutte JE, Longhurst JC, Gaffney FA, Bastian BC, Blomqvist CG (1981). Total plasma creatinine: An accurate measure of total striated muscle mass. J. Appl. Physiol. Respir. Environ. Exerc. Physiol..

[17] Cabinet Office Japan. Annual Report on the Ageing Society [Summary] FY 2019. (Cabinet Office Japan, 2019).

[18] Sugimoto K (2019). Hyperglycemia in non-obese patients with type 2 diabetes is associated with low muscle mass: The multicenter study for clarifying evidence for sarcopenia in patients with diabetes mellitus. J. Diabetes Investig..

[19] Hirata Y (2019). Hyperglycemia induces skeletal muscle atrophy via a WWP1/KLF15 axis. JCI Insight.

[20] Harita N (2009). Lower serum creatinine is a new risk factor of type 2 diabetes: The Kansai healthcare study. Diabetes Care.

[21] Kashima S, Inoue K, Matsumoto M, Akimoto K (2017). Low serum creatinine is a type 2 diabetes risk factor in men and women: The Yuport Health Checkup Center cohort study. Diabetes Metab..

[22] Ministry of Health, Labour, and Welfare, Japan. *The National Health and Nutrition Survey in Japan, 2017.* https://www.mhlw.go.jp/content/000451755.pdf (2019).

[23] Rius F (1995). Age as a determinant of glomerular filtration rate in non-insulin-dependent diabetes mellitus. Nephrol. Dial. Transplant..

[24] Premaratne E (2005). Renal hyperfiltration in type 2 diabetes: Effect of age-related decline in glomerular filtration rate. Diabetologia.

[25] Park J (2013). Serum creatinine level, a surrogate of muscle mass, predicts mortality in peritoneal dialysis patients. Nephrol. Dial. Transplant..

[26] Thongprayoon C, Cheungpasitporn W, Kashani K (2016). Serum creatinine level, a surrogate of muscle mass, predicts mortality in critically ill patients. J. Thorac. Dis..

[27] Thongprayoon C (2015). Optimum methodology for estimating baseline serum creatinine for the acute kidney injury classification. Nephrology.

[28] Inoue K, Matsumoto M, Miyoshi Y, Kobayashi Y (2008). Elevated liver enzymes in women with a family history of diabetes. Diabetes Res. Clin. Pract..

[29] International Expert Committee (2009). International Expert Committee report on the role of the A1C assay in the diagnosis of diabetes. Diabetes Care.

[30] Seino Y (2010). Report of the committee on the classification and diagnostic criteria of diabetes mellitus. Diabetol. Int..

[31] Kashiwagi A (2012). International clinical harmonization of glycated hemoglobin in Japan: From Japan Diabetes Society to National Glycohemoglobin Standardization Program values. J. Diabetes Investig..

[32] Zelen M (1971). The analysis of several 2× 2 contingency tables. Biometrika.

